# Improvement of the Biosynthesis of Resveratrol in Endophytic Fungus (*Alternaria* sp. MG1) by the Synergistic Effect of UV Light and Oligomeric Proanthocyanidins

**DOI:** 10.3389/fmicb.2021.770734

**Published:** 2021-10-21

**Authors:** Yao Lu, Junling Shi, Xixi Zhao, Yuyang Song, Yi Qin, Yanlin Liu

**Affiliations:** ^1^College of Enology, Northwest A&F University, Yangling, China; ^2^Ningxia Helan Mountain’s East Foothill Wine Experiment and Demonstration Station of Northwest A&F University, Yongning, China; ^3^Key Laboratory for Space Bioscience and Biotechnology, School of Life Sciences, Northwestern Polytechnical University, Xi’an, China

**Keywords:** resveratrol, endophytic fungus, *Alternaria*, UV light, oligomeric proanthocyanidins

## Abstract

Resveratrol, a natural polyphenol compound with multiple bioactivities, is widely used in food and pharmaceutical industry. Endophytic fungus *Alternaria* sp. MG1, as a native producer of resveratrol, shows increasing potential application. However, strategies for improvement of the biosynthesis of resveratrol in this species are still scarce. In this study, different elicitors were used to investigate their effect on the biosynthesis of resveratrol in MG1 and the induction mechanism. Ultrasound and sodium butyrate had no effect and slight inhibition on the resveratrol production and related gene expression, respectively. UV radiation and co-culture with *Phomopsis* sp. XP-8 significantly promoted the biosynthesis of resveratrol with the highest production (240.57μg/l) coming from UV 20min. Co-culture altered the profiles of secondary metabolites in MG1 by promoting and inhibiting the synthesis of stilbene and lignin compounds, respectively, and generating new flavonoids ((+/−)-taxifolin, naringin, and (+)-catechin). Oligomeric proanthocyanidins (OPC) also showed an obviously positive influence, leading to an increase in resveratrol production by 10 to 60%. Two calcium-dependent protein kinases (CDPK) were identified, of which CDPK1 was found to be an important regulatory factor of OPC induction. Synergistic treatment of UV 20min and 100μm OPC increased the production of resveratrol by 70.37% compared to control and finally reached 276.31μg/l.

## Introduction

Resveratrol is a type of natural polyphenol compound that is widely found in diverse plants, fruits, red wine, and even endophytic fungi. Resveratrol has a broad spectrum of biological and pharmacological properties due to its ability to regulate many signaling pathways (Ahmadi and Ebrahimzadeh, 2020). Extensive research has shown that resveratrol can prevent and treat many diseases, including cancer, diabetes, cardiomyopathy, senescence, pneumonia, and acute respiratory distress (Filardo et al., 2020). A new study suggests that resveratrol is expected to be a novel drug for preventing and alleviating the symptoms of COVID-19 (Ferreira et al., 2020). This assumption is based on that it can activate NK cells, suppress the expression of TLR4 and pro-inflammatory factors (TNF-α, IL-1β, and IL-6), and decrease the expression of metalloproteinases (MMP-1 and MMP3) and Cox-2 induced by the transcription factor NF-kB. In addition, resveratrol presents potential to reduce virus entrance in the cells and synergize with zinc to reduce virus replication rate (Cheng et al., 2020). Resveratrol also shows great potential in the food and cosmetics industry. It can be used as an additive in yogurt or as a component of skin care and beauty products because of its antioxidant, whitening, and anti-acne activities (Pando et al., 2015; Ratz-Łyko and Arct, 2019). In the field of biosynthesis, resveratrol is important precursor for its analogues. It can be converted to pterostilbene and piceatannol by trans-resveratrol di-*O*-methyltransferase (ROMT, 2.1.1.240) and the cytochrome P450 enzyme (CYP1B1), respectively (Lu et al., 2019). Pterostilbene and piceatannol are considered to have stronger antioxidant, anti-cancer, and antifungal properties compared with resveratrol (Marco et al., 2000; Zdenka et al., 2006). Significantly, pterostilbene has been approved by the FDA to be recognized as safe (GRAS) status as a food ingredient, which provides new commercial opportunities in natural food and beverage processing (Wang et al., 2018). Therefore, the high demand for resveratrol keeps growing because of its high medicinal and dietary value.

In nature, resveratrol is normally produced by plants *via* the phenylpropanoid pathway (PPPN). The previous study revealed that resveratrol in endophytic fungi was also synthesized through this pathway (Lu et al., 2019). Basically, PPPN begins with the synthesis of phenylpropanoic acids from aromatic amino acid phenylalanine (Phe) or tyrosine (Tyr) that derived from shikimate pathway (Lim et al., 2011). In this step, there are two branches. Tyrosine ammonia-lyase (TAL) catalyzes the desamination reaction of Tyr to form *p*-coumaric acid. Next, 4-coumarate coenzyme A ligase (4CL) combines *p*-coumaric acid and coenzyme A (CoA) to produce 4-coumaroyl-CoA. In the separate branch, cinnamic aid is formed as the product of Phe catalyzed by phenylalanine ammonia-lyase (PAL) (Mei et al., 2015). Cinnamic acid is then continuously catalyzed by cinnamate 4-hydroxylase (C4H) and 4CL in either order to form 4-coumaroyl-CoA. Finally, one molecule of 4-coumaroyl-CoA and three molecules of malonyl-CoA are catalyzed by stilbene synthase (STS) to form resveratrol. STS belongs to type III polyketide synthases (PKSs); in addition, PKSs also includes chalcone synthase (CHS). It is noteworthy that, although CHS is responsible for the biosynthesis of naringenin, cross-reactivity between CHS and STS has been demonstrated with CHS forming resveratrol and STS forming naringenin (Yamaguchi et al., 1999). Therefore, *PAL*/*TAL*, *4CL*, *C4H*, and *STS*/*CHS* are key genes involved in the synthesis of resveratrol.

Plant extraction is a traditional method to produce resveratrol, but there are some disadvantages, such as low yield, long growth period of plants, and the need for stimulation by biotic and/or abiotic stress conditions (Wang et al., 2018). Chemical synthesis of resveratrol requires a complex procedure and the use of toxic organic solvents and also generates unwanted byproducts (Lu et al., 2019). Microbial fermentation with engineered microorganisms, like *Saccharomyces cerevisiae*, *Escherichia coli*, and *Yarrowia lipolytica*, is one of the most promising and eco-friendly approach for the scale-up production of resveratrol (Lu et al., 2016). However, metabolic engineering by introducing or knockout of specific genes is expensive and time-consuming. Recently, endophytic fungi exhibit significant potential for producing plant-original drugs and functional compounds, such as taxol (Venugopalan and Srivastava, 2015). Endophytic fungi acquired the ability to synthesize secondary metabolites similar to their host plants during the evolutionary process of symbiosis with plants. In addition, these microorganisms showed advantages of accumulating high concentrations of products that would be toxic to genetically modified *E. coli* and yeast since they possess high resistance to their self-produced metabolites (Kusari et al., 2011). Up to now, many resveratrol-producing endophytic fungi have been isolated and identified, mainly including *Aspergillus*, *Botryosphaeria, Penicillium*, *Fusarium*, *Alternaria*, *Arcopilus*, and *Lasiodiplodia*. Also, resveratrol has been detected in macro fungi, such as wild edible mushrooms (*Pleurotus eryngii*, *Lactarius deliciosus*, *Russula delica*, *Suillus bellinii*, etc.) (Yildirim et al., 2012; Nick et al., 2013) and medicinal mushrooms (*Sparassis crispa*, *Inonotus obliquus*) (Min-Young et al., 2008), and even transgenic mushroom *Flammulina velutipes* (Kang et al., 2014).

*Alternaria* sp. MG1 is an endophytic fungus previously isolated from the cob of *Vitis vinifera* L. cv. Merlot that could stably produce resveratrol using glucose as a substrate (Shi et al., 2012). However, the production of resveratrol by MG1 was low which hinders it from being an industrial resveratrol producer. Generally, the accumulation of secondary metabolites in plants is associated with their continuous response to various biotic and/or abiotic stresses. Considering that endophytic fungi have similar secondary metabolic characteristics with their plant hosts, it is possible that endophytic fungi may also accumulate secondary metabolites in this way. For example, an endophyte from Yew trees synthesized taxol to defend its host plant against wood-decaying fungi through the formation of extracellular barriers (Soliman et al., 2015). Therefore, the present study focuses on the effects of biotic (*Phomopsis* sp. XP-8) and abiotic [ultrasound, UV light, sodium butyrate, and oligomeric proanthocyanidins (OPC)] elicitors on the production of resveratrol and resveratrol biosynthesis genes. The induction mechanism of some specific elicitors was also investigated. The study will shed light on the importance of elicitors in improvement of resveratrol yield in endophytic fungi and provide useful information for further research on developing MG1 as an alternative source of producing resveratrol and other secondary metabolites.

## Materials and Methods

### Microorganisms, Medium, and Cultivation

*Alternaria* sp. MG1 (code: CCTCC M 2011348) and *Phomopsis* sp. XP-8 (code: CCTCC M 209291), currently preserved in the China Centre for Type Culture Collection (Wuhan, China), were used in the study. *Phomopsis* sp. XP-8 is an endophytic fungus isolated from the bark of Tu-chung (*Eucommia ulmoides* Oliv.) by our group that produces lignins, including (+)-pinoresinol, (+)-pinoresinol monoglucoside, and (+)-pinoresinol diglucoside. The strains were cultivated in potato-dextrose broth (PDB) at 28°C and 160rpm for 7days. PDB medium was prepared with 20g potato, 2g dextrose, and 100ml distilled water.

### Effect of Biotic Elicitor on the Resveratrol Biosynthesis

The spore suspension (1×10^7^ spores/ml) of strain MG1 and XP-8 was separately prepared (Zhang et al., 2013) and co-cultured in PDB medium at the inoculation ratio of 5% each. After cultivation for 7days, the cells were collected from the culture by vacuum filtration and used for analysis of key genes expression. The cell-free cultures were then used for measurement of resveratrol production and the analysis of metabolite profile. For the control group, only 5% of MG1 or XP-8 was inoculated, and 3 replicates were set for each treatment.

### Effect of Abiotic Elicitors on the Resveratrol Biosynthesis

Abiotic elicitors included physical and chemical factors. For physical induction, the 4-day culture of MG1 was separately treated with ultrasound (40kHz, 10min) and UV light (350nm; 10, 20, 30, 40min). For chemical induction, sodium butyrate was filtered through a 0.22μm hydrophilic membrane and then added to the 4-day culture of MG1 at final concentration of 100μm. OPC was kindly provided by Prof. Dongyan Shao (School of Life Sciences, Northwestern Polytechnical University). It was dissolved in dimethyl sulfoxide (DMSO) and added to the 4-day culture of MG1 at different final concentrations of 50, 100, 150, and 200μm of 1ml. To make sure whether the effect of OPC on the resveratrol biosynthesis is related to the solvent in which it is dissolved, 1ml of DMSO solution was also added to the MG1 culture as a separate treatment group. All treated cultures of MG1 were continuously cultivated to the 7th day. Then, cells were collected and stored for analysis of key genes expression. The cell-free cultures were used for the measurement of resveratrol production. Each treatment was performed in triplicate, and samples without elicitor treatment were always run in parallel as control.

### Quantitative Analysis of Resveratrol Using HPLC

Quantitative analysis of resveratrol was performed using HPLC (Water 2,695 system, Alliance, MA, United States) equipped with a WondaSil C18-WR column (5μm, 4.6mm×250mm, GL Sciences Inc., Japan). The cell-free cultures were extracted with equal volume of ethyl acetate three times (10h each time). The ethyl acetate phase was collected and vacuum concentrated in a rotary evaporator (Changzheng Instrument Manufacturing Co., Ltd., Zhengzhou, China) to dryness at 40°C. Then, the dried residue was dissolved in 2ml chromatographic grade methanol and filtered through a 0.22μm membrane (Solarbio, Beijing, China). The detection conditions for resveratrol were the same as that described before (Lu et al., 2019). The column was operated at 35°C. The mobile phase consisted of A: acetonitrile, B: ddH_2_O. A multi-step gradient was used at a flow rate of 1ml/min according to the following steps: 0–28min, A: 5%, B: 95%; 28–33min, A: 60%, B: 40%; 33–40min, A: 85%, B: 15%; 40–50min, A: 5%, B: 95%. The detection wavelength was 306nm. The injection volume was 20μl.

### qRT-PCR Analysis of Key Genes Expression

Samples were prepared from 7-day growing cells with or without elicitor treatments. Total RNA was extracted, and cDNA was synthesized using commercial kits (Sangon Biotech Co., Ltd., Shanghai, China; Transgene Biotech Co., Ltd., Beijing, China). Primers of target genes are shown in Table 1. The reaction volume was set to 20μl in accordance with the operation manual of the TransStart Tip Green qPCR SuperMix Kit (Transgene Biotech Co., Ltd., Beijing, China). All reactions were conducted in triplicate. The program and qRT-PCR analysis were performed as previously described (Livak and Schmittgen, 2002). For each gene, the expression value was normalized with respect to the reference genes *EF1* and *TUBA* and was calculated *via* 2^-ΔΔCT^ method (Che et al., 2016).

**Table 1:** Primer sequences used for the analysis of expression level of target genes.

Gene	Definition	Primer sequence
*EF1*	Elongation factor 1	F 5'-CACTGGTTTTGCCTTTTCCT-3'
		R 5'-TGTGGGCACCGTCAAAGT-3'
*TUBA*	α-Tubulin	F 5'-CAAGCGAGTCAGAAGC-3'
		R 5'-GGTATGTTGGTGAGGGTAT-3'
*PAL*	Phenylalanine ammonia-lyase	F 5'-CTGGGAACTGACCAAGCG-3'
		R 5'-ATGAGGAGGTATCCGAGCC-3'
*C4H*	Cinnamate-4-hydroxylase	F 5'-GTGGAGTCACGGGAAGTAAG-3'
		R 5'-TGAAGGCAACCCTCAAGAT-3'
*4CL*	4-Coumaryl-CoA synthetase	F 5'-GGTGGCTTGAATGTGAAT-3'
		R 5'-CAACTACTCGTCGGGAAC-3'
*CHS*	Chalcone synthase	F 5'-CTCACTATCACCGCCTTCC-3'
		R 5'-CAGCACCCACGATGACG-3'
*CDPK1*	Calcium-dependent protein kinase 1	F 5'-TTGACTGGTGAAACGGAGAAGCT-3'
		R 5'-TGGAACTGGCAGACAACGTAGGG-3'
*CDPK2*	Calcium-dependent protein kinase 2	F 5'-ATGACGACGACCTTCCCGTGCTG-3'
		R 5'-CAAATGGTATGGACTTTGTTGTGGC-3'

### Analysis of Metabolite Profiles Using GC–MS

#### Metabolite Extraction and Derivatization

The cell-free culture was freeze-dried into powder and ground. Then, samples were extracted with 0.5ml extraction liquid of a mixture of methanol and chloroform (*V: V*=3: 1) in the 2-ml EP tubes. A 20μl of L-2-Chlorophenylalanine (1mg/ml stock in dH_2_O) was also added as an internal standard to the sample. After homogenizing in ball mill (MM 400, Retsch, Germany) for 4min at 45Hz, samples were incubated in ice water and ultrasound treated for 5min and then centrifuged by a Primo R Centrifuge (Thermo Scientific, Waltham, MA, United States) for 15min (13,000rpm, 4°C). The supernatant (0.4ml) was transferred into a fresh 2ml GC/MS glass vial and dried in a vacuum concentrator without heating. Methoxy amination hydrochloride (20mg/ml in pyridine, 80μl) was added to the dried samples, and they were incubated at 80°C for 30min. Then, 100μl of BSTFA reagent with 1% trimethylsilyl chloride was added, and samples were incubated for 2h at 70°C before GC–MS analysis.

#### GC–MS Analysis

GC-TOFMS analysis was performed using an Agilent 7,890 gas chromatograph system coupled with a Pegasus HT time-of-flight mass spectrometer. The system utilized a DB-5MS capillary column-coated with 5% diphenyl cross-linked with 95% dimethylpolysiloxane (30m×250μm inner diameter, 0.25μm film thickness; J&W Scientific, Folsom, CA, United States). A 1μl aliquot of the analyte was injected in splitless mode. Helium was used as the carrier gas, the front inlet purge flow was 3ml/min, and the gas flow rate through the column was 1ml/min. The initial temperature was kept at 50°C for 1min, then raised to 310°C at a rate of 10°C min^−1^ and then kept for 8min at 310°C. The injection, transfer line, and ion source temperatures were 280, 270, and 220°C, respectively. The energy was −70eV in electron impact mode. The mass spectrometry data were acquired in full-scan mode with the m/z range of 50–500 at a rate of 20 spectra per second after a solvent delay of 455s.

#### Data Processing

Chroma TOF 4.3X software and LECO-Fiehn Rtx5 database were used for raw peaks extracting, the data baselines filtering and calibration of the baseline, peak alignment, deconvolution analysis, peak identification, and integration of the peak area (Kind et al., 2009).

### Identification of CDPK

The published sequences of different CDPK proteins were chosen from UniProt database for multiple sequence alignment using the DNAMAN software package (version 7.0.2, LynnonBiosoft Company, United States). A phylogenetic tree was constructed using MEGA (Molecular Evolutionary Genetics Analysis) software version 7.0 with 1,000 bootstrap replicates. Sequences of the identified CDPK genes in MG1 are shown in Supplementary File 1.

### Statistical Analysis

All chemical data were presented as means ± standard deviation (SD) from triplicate determinations and processed with Origin 8.5. Multiple comparison analysis was performed by Duncan test using IBM SPSS Statistics 22.0 (SPSS Inc., Chicago, IL, United States). The significance level was set at *p*<0.05.

## Results and Discussion

### Effect of Different Elicitors on the Biosynthesis of Resveratrol in *Alternaria* sp. MG1

**Figure 1:**
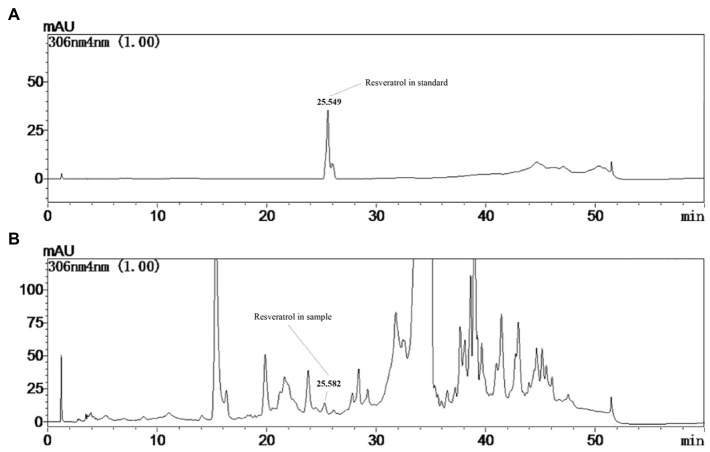
Determination of resveratrol in the culture of *Alternaria* sp. MG1. HPLC chromatogram of resveratrol standard **(A)**. HPLC chromatogram of the resveratrol detected in control group of MG1 **(B)**.

**Figure 2:**
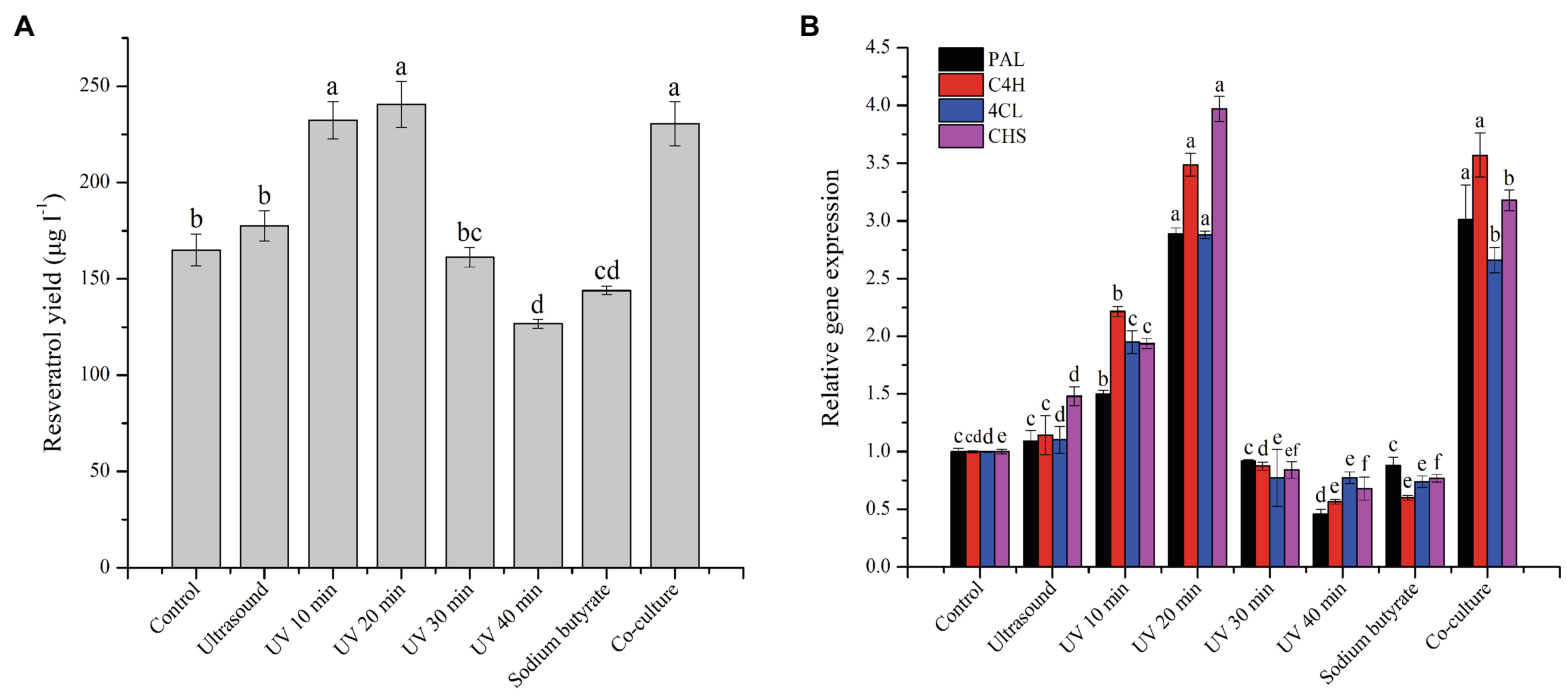
Effect of different elicitors on the resveratrol production **(A)** and the expression of key genes in the pathway **(B)**. Values with different letters indicate a significant difference (Duncan, *p*<0.05).

The production of resveratrol was detected at the seventh day under the controlled conditions using PDB medium at 28°C. From Figure 1, a product having the same retention time (25.582) as that of standard resveratrol (25.549), and its content was calculated to be around 160μg/l. To improve the production of resveratrol in MG1, different elicitors were selected and investigated. The results showed that ultrasound treatment did not significantly increase the production of resveratrol, but sodium butyrate had an inhibitory effect on its production. Co-culture with *Phomopsis* sp. XP-8 exhibited an obvious effect on the biosynthesis of resveratrol, which increased its production by approximately 40% (Figure 2A). In addition, the resveratrol production increased first and then decreased rapidly with the extension of UV irradiation time. It reached the highest value of 240.57μg/l at 20min, which increased by 45.8% compared with the control. The effect of different elicitors on the expression of key genes was also analyzed. In our previous study, a total of 5 *PAL*, 1 *C4H*, 6 *4CL*, and 1 *CHS* genes involved in the biosynthesis pathway of resveratrol were annotated in MG1 by genomic sequencing (Lu et al., 2019). Interestingly, no STS gene was annotated. Among these genes, *CHS* (Accession number: KX197069.1) was identified to synthesize both naringenin and resveratrol, while one of *4CL* genes (Accession number: KX179648.1) synthesized *p*-coumaroyl CoA (Lu et al., 2021). Here, representative genes that have been functionally validated and those that are being functionally validated were selected for study. The results showed that under the same treatment condition, its effect on key genes expression was consistent with the trend of its effect on the production of resveratrol (Figure 2B). Among them, UV irradiation for 20min or co-culture with XP-8 significantly upregulated the expression of key genes of the resveratrol pathway, which was about 3–4 times that of the control.

Ultrasound is well-known reported to influence the biosynthesis efficiency of metabolites, growth rates, and enzymatic activity by improvement of cell permeability as well as mass transfer and nutrient uptake rates through cell membranes (Behzadnia et al., 2020). Valente et al. found that the antioxidant activity of exocellular metabolites of *Botryosphaeria dothidea* after ultrasound treatment was higher than the control, reaching a maximum value of 96% (Valente et al., 2020). In addition, mild intensity ultrasound (low energy and short exposure time) could significantly promote anticoagulant accumulation by marine *Bacillus subtilis* ZHX (Cao et al., 2020) and mycelial growth and exopolysaccharides (EPS) biosynthesis from *Agaricus bitorquis* (Quél.) Sacc. Chaidam (Lu et al., 2020). In the current study, ultrasound exposure (40kHz) for 10min caused no significant increase of resveratrol. This might be because resveratrol biosynthesis of MG1 is not sensitive to ultrasound disturbance. Similar result could be also found in the case of production of bioactive valepotriates in *Valeriana glechomifolia* (Russowski et al., 2013). Sodium butyrate is widely reported to show eliciting activity as a histone deacetylases inhibitor, which enables silent or cryptic gene clusters to express. The yield of enzymes and secondary metabolites from fungi could be increased, and new metabolites like phenolic compounds are generated after sodium butyrate treatment (El-Hawary et al., 2018; Mohammadipanah et al., 2020). However, 100μm of sodium butyrate had an adverse influence on the biosynthesis of resveratrol in MG1, which may be related to the inhibition of fungal growth by sodium butyrate. The biomass of some fungi decreased significantly after being treated with sodium butyrate, such as *Phomopsis* and *Trichosporon* spp. (Zhu et al., 2018; Cordeiro et al., 2019). Therefore, it is speculated that 100μm of sodium butyrate used in this study inhibited the biomass of MG1, which lead to insufficient supply of some important precursors of resveratrol, such as phenylalanine and tyrosine. It is noteworthy that the elicitor potential of sodium butyrate could be concentration-dependent (Fatemeh et al., 2020). Therefore, the failure to elicit the production of resveratrol at a single concentration of 100μm did not mean that its production under other concentrations or the production of other metabolites was not affected. A further dose–response experiment is required regarding the potency of this elicitor. UV radiation is an important mutagen for high-yielding strains. For example, strain *Cladosporium phlei* M0035 resulted in a total of 592mg/l phleichrome after UV-mutagenesis, more than seven-fold over wild type (Yi et al., 2011). Similarly, the highest amount of monacolin K produced by the mutated strain of *Monascus* spp. was 3 times greater than the control after the irradiation of UV light in the presence of 1.0‰ LiCl in the medium (Sun et al., 2011). In addition, UV radiation is also commonly used to increase the production rate and the catalytic activity of enzymes of fungi. The production of resveratrol synthesized by MG1 gradually increased with the increase of UV treatment time, which may be a self-adaptive protection to adverse environments. When the treatment time increased to 30min or even longer, it may damage the cell structure and enzyme activity, resulting in the inhibition of resveratrol biosynthesis.

### Effect of Co-culture With *Phomopsis* sp. XP-8 on the Metabolite Profile of MG1

**Figure 3:**
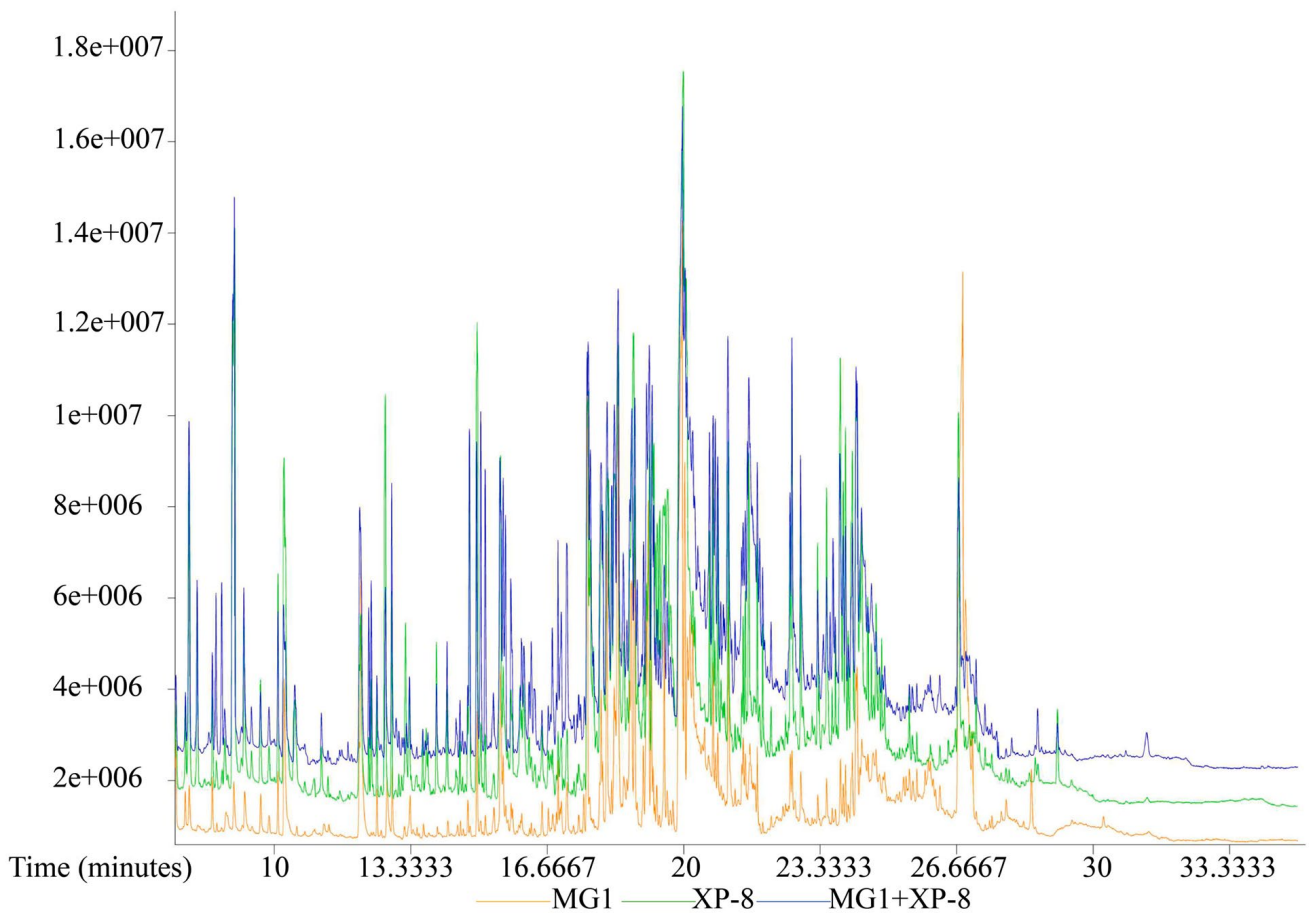
Representative GC–MS TIC chromatograms of MG1 culture, co-culture (MG1 + XP-8), and XP-8 culture.

In nature, fungi lives in association with other organisms and this constant interaction affect the quality and quantity of secondary metabolites produced. Co-culture of different bacteria, bacteria and fungi as well as different fungi shows great advantages in improving the production of natural products with pharmaceutical properties, because they have similar pathways and could provide each other with complementary key genes for their biosynthesis. Co-cultivation of *Paraconiothyrium* SSM001 and *Alternaria* increased the production of taxol in SSM001 by 3 times (Soliman and Raizada, 2013). Similar studies could also be found in the enhancement of secondary metabolites subenniatins A and B, fusaristatin A, podophyllotoxins, and stemphyperylenol (Baldi et al., 2010; Chagas et al., 2013; Ola et al., 2013; Wang et al., 2013). Strains *Alternaria* sp. MG1 and *Phomopsis* sp. XP-8 are both plant endophytic fungi that isolated by our group in the previous study, so they were used to investigate the effect of co-culture system on the biosynthesis of resveratrol in MG1. The production of resveratrol in MG1 increased by 40% when co-cultured with XP-8, revealing microbial interaction may induce the expression of related silent gene clusters. Therefore, the metabolite composition of the culture broth from MG1 was analyzed by GC–MS to further clarify the effect of co-culture with XP-8 on the metabolite profile of MG1. As shown in Figure 3, the chromatographic peaks of different components exhibited good separation, and most of the peaks were well separated from the baseline. A total of 511, 1,458, and 1,535 distinct peaks were detected in the chromatograms for the culture broth of MG1, co-culture of MG1 and XP-8 (MG1 + XP-8), and XP-8, respectively. Then, after preprocessing of raw peaks with Chroma TOF software, strict quality control procedures, and peak identification, a total of 379, 832, and 875 metabolites were identified with a similarity above 200 in MG1, MG1 + XP-8, and XP-8, respectively.

As MG1 and XP-8 are both plant endophytic fungi and have similar metabolic pathways, we used the method of peak area normalization to only quantify the relative contents of unique secondary metabolites (from stilbene and flavonoid biosynthesis pathways) in MG1. As shown in Table 2, after co-culture with XP-8, the accumulation of resveratrol and piceatannol in MG1 increased, which was consistent with the above results, while naringenin, coniferyl alcohol, and sinapic acid were not detected compared with before co-culture. This indicated that co-culture promoted the synthesis of stilbene compounds in MG1 but completely inhibited the formation of naringenin and lignin compounds.

**Table 2:** Relative content of specific secondary metabolites of MG1.

Chemical name	RT/min	Formula	Content^a^	Content^b^
Resveratrol	24.1583	C14H12O3	0.0413	0.0694
Piceatannol	27.5325	C14H12O4	0.0087	0.0134
Naringenin	28.3933	C15H12O5	0.0026	NA
Coniferyl alcohol	20.1625	C10H12O3	0.0134	NA
Sinapic acid	21.1475	C11H12O5	0.0038	NA

a *Relative content of metabolite in the culture broth of MG1*.

b *Relative content of metabolite in the culture broth of MG1 + XP-8, NA not detected, RT retention time*.

**Figure 4:**
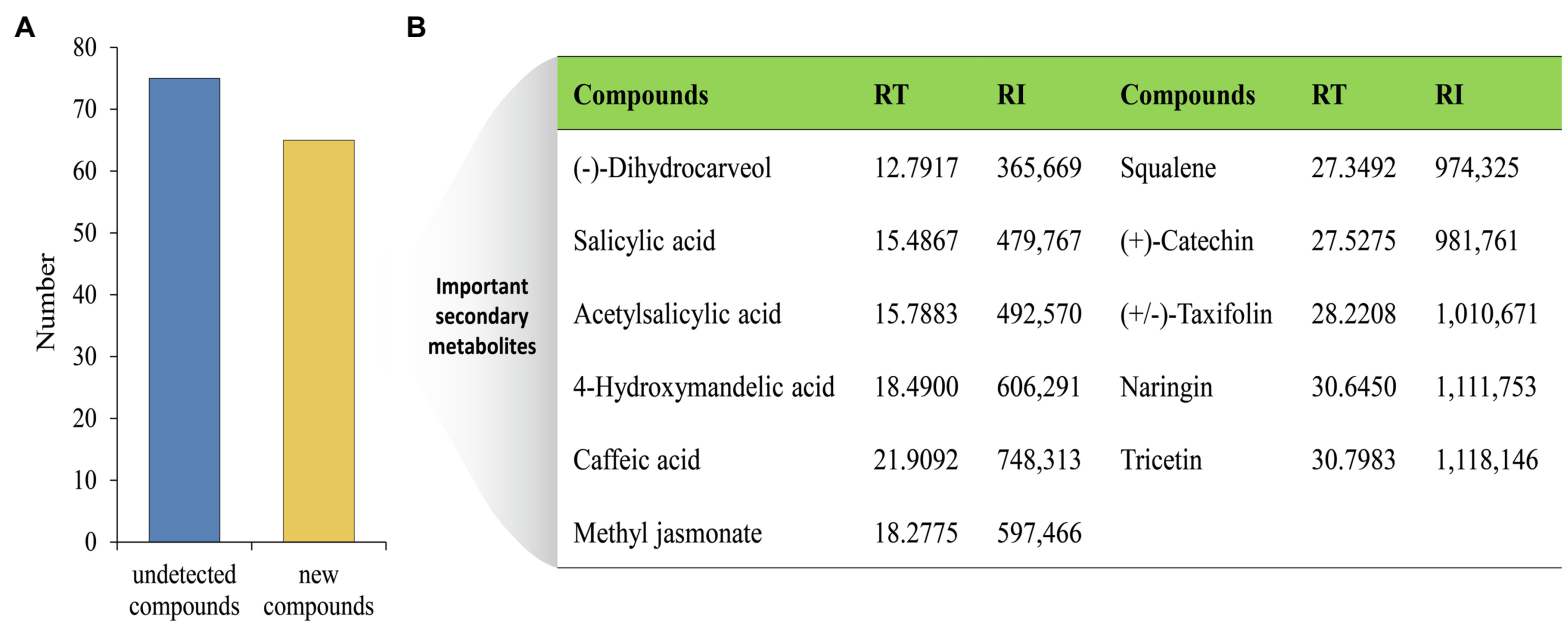
Metabolites changes in MG1 after co-cultured with XP-8. The number of undetected and new compounds in MG1 after co-culture **(A)**. Important secondary metabolites selected from new generated compounds in MG1 after co-culture **(B)**. RT, retention time; RI, retention index.

The changes of metabolites in MG1 culture broth after co-cultivation were further analyzed. A total of 65 new compounds were generated (Figure 4A), among which the more important secondary metabolites included: (−)-dihydrocarveol, salicylic acid, acetylsalicylic acid, 4-hydroxymandelic acid, caffeic acid, squalene, (+)-catechin, (+/−)-taxifolin, naringin, tricetin, and methyl jasmonate (Figure 4B). These chemicals exhibit a variety of biological activities and are widely used as fragrances, antioxidants, and drugs, etc. It is worth noting that (+/−)-taxifolin, naringin, and (+)-catechin are all belong to flavonoids. However, as mentioned above, their common precursor naringenin in MG1 was not detected after co-cultured with XP-8, indicating that the synthesis of naringenin was not actually inhibited, but was completely converted to different flavonoid compounds after co-culture. The results showed that co-culture with XP-8 promoted the further transformation of naringenin in MG1. Moreover, a total of 75 compounds were not detected in MG1 after co-cultivation with XP-8. This phenomenon, the complete bioconversion of a certain metabolite induced by co-culture, is commonly found in other studies. Eudistomin I is a B-carboline alkaloid, and its synthesis begins with tryptamine as a starting material. Rafidaf et al. (2020) found that the tryptamine that was detected in the control group was used up by the co-culture fungi and involved in the biosynthetic pathway to produce eudistomin I, which explains why tryptamine was not detected in the treatment group. In addition, three O-glycosylated isomers of naringenin dropped, with two of these reaching non detectable levels in okara fermented with co-culture strains of lactic acid bacteria (Jasmine et al., 2021). However, the conclusion needs to be supported by investigating whether the expression of genes involved in the synthesis of downstream flavonoids in MG1 is upregulated.

### Effect of OPC on the Biosynthesis of Resveratrol in *Alternaria* sp. MG1

**Figure 5:**
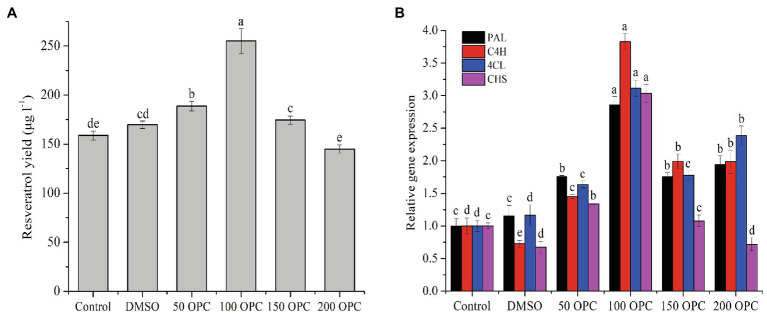
Effect of OPC on the resveratrol production **(A)** and the expression of key genes **(B)**. Values with different letters indicate a significant difference (Duncan, *p*<0.05).

Proanthocyanidin is an important nutrient component in grape fruit and wine, and its existing form is mainly OPC. Endophytic fungi parasitizing on grapes (like skin, cob, and stem) inevitably enter the fermentation process with the crushing and pressing of grape raw materials, and their growth and physiological metabolism are affected by the main components of the wine. Therefore, in order to explore the influence of OPC on the biosynthesis of resveratrol in MG1, different concentrations of OPC were used to treat the MG1 cells. As shown in Figure 5A, the response of resveratrol biosynthesis to OPC treatment was dose-dependent. That was, its production increased with the increase of OPC concentration and reached the highest value (255.01μg/l) at 100μm of OPC, which was an improvement of 60.63% compared to the control, while its production decreased gradually when the OPC concentration was higher than 100μm and was significantly inhibited at 200μm of OPC. The effect of OPC treatment on the expression of key genes in the resveratrol pathway was basically consistent with the trend of its effect on the resveratrol yield (Figure 5B). However, at 200μm of OPC, the expression levels of *PAL*, *C4H*, and *4CL* were all significantly upregulated, about 2–2.5 times that of the control, while the expression level of *CHS* was obviously downregulated. This indicated that the reaction catalyzed by the enzyme encoded by *CHS* gene was the rate-limiting step of the resveratrol pathway, leading to no significant increase in its yield. This conclusion was also valid with the corresponding relationship between the expression of *CHS* gene and resveratrol production under other OPC concentration conditions.

**Figure 6:**
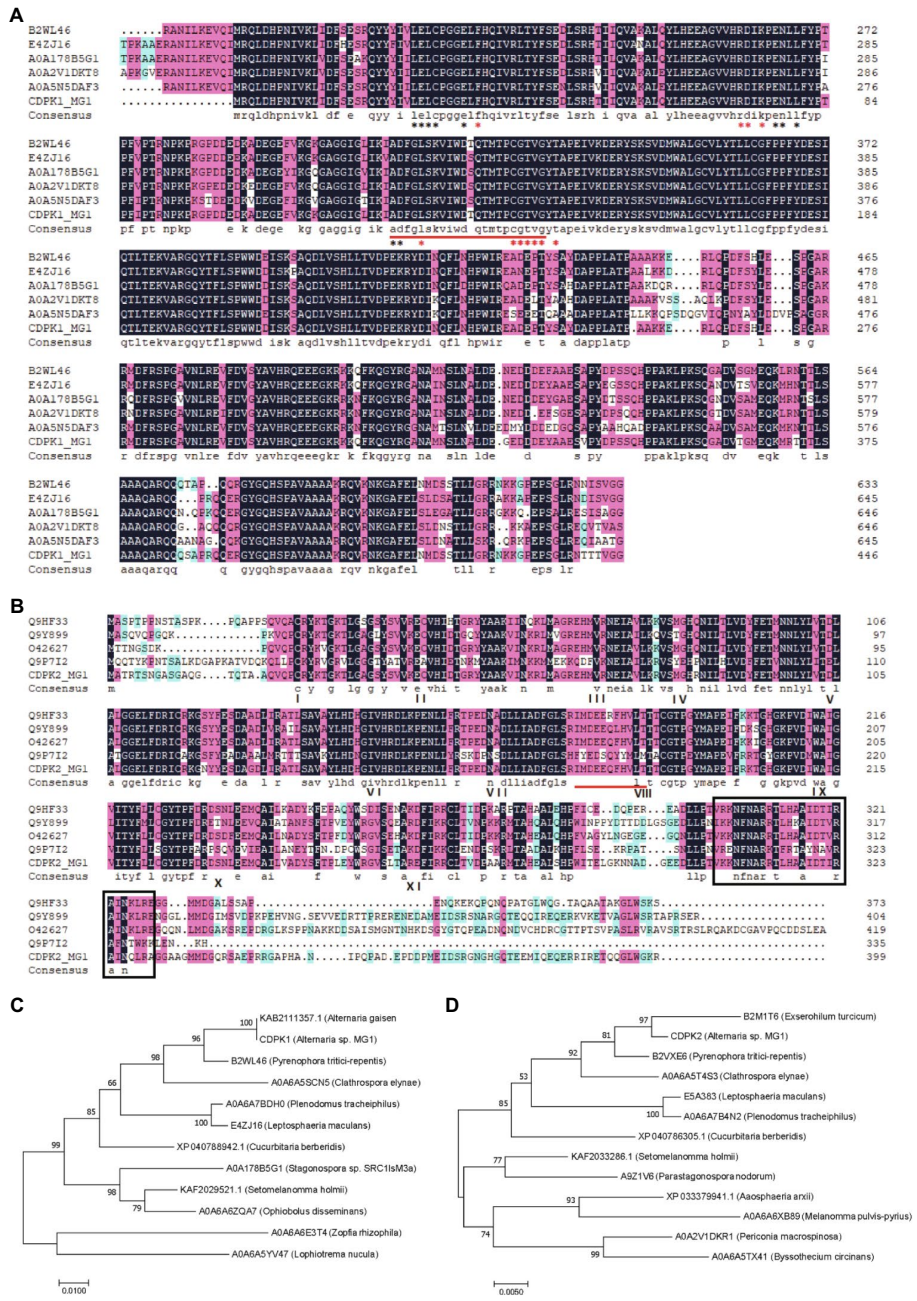
Sequence analyses of CDPK proteins. Multiple sequence alignment of CDPK1 **(A)** and CDPK2 **(B)** with other calmodulin-dependent proteins. The putative activation loop motifs are indicated by red underline. The ATP binding site and polypeptide substrate binding site are marked with black and red asterisks, respectively. The numbers I-XI below the CDPK2 sequence indicate conserved protein kinase catalytic subdomains. The calmodulin-binding domain is highlighted with black border. Identical amino acids are shown with dark blue backgrounds, highly similar (50–99%) amino acids are shown with pink backgrounds, and amino acids with low similarity (33–50%) are shown with light blue backgrounds. Phylogenetic analysis of CDPK1 **(C)** and CDPK2 **(D)** with other CDPK sequences as a neighbor-joining tree.

Many plant-derived secondary metabolites have been reported to react with protein kinases involved in the signal transduction of eukaryotic cell, thereby causing a series of biological responses (Molan et al., 2000). Ca^2+^ is an important second messenger involved in plant cell signal transduction. When plants are exposed to external stresses, the Ca^2+^ signals in the cell are transmitted to the downstream components through Ca^2+^ sensors to elicit the expression level of related genes, so as to respond to various environmental changes (Romeis and Herde, 2014). Calcium-dependent protein kinase (CDPK) is one of the important proteins involved in the regulation of Ca^2+^ and was found to cause significant accumulation of resveratrol when overexpressed in transgenic plant cells (Aleynova-Shumakova et al., 2014). CDPK proteins (CDPKs) mediate various Ca^2+^ signalings through CDPK cascades in response to various extracellular stimuli (Isamu et al., 2007). Therefore, as an important plant-derived secondary metabolite, whether OPC can activate Ca^2+^ channels by interacting with Ca^2+^ signal-related proteins, such as CDPK proteins, thereby activating the upregulation of downstream component (resveratrol) and related genes to resist stress environment caused by OPC, are not clear. Moreover, it is unclear whether there are CDPK proteins in MG1. To uncover the possible regulatory mechanism between OPC, CDPK protein and resveratrol biosynthesis in MG1, the expression level of CDPK protein under different OPC concentrations was investigated. Through mining the genomic data of MG1, two *CDPK* genes were found, named *CDPK1* and *CDPK2* (Supplementary File 1). The *CDPK1* sequence consisted of 1,344bp encoding 447 amino acid resides and *CDPK2* sequence consisted of 1,200bp encoding 399 amino acid resides. According to the results of multiple sequence alignment, two CDPK proteins from MG1 had high identity with other reported CDPKs from different species, among which CDPK1 and CDPK2 showed the identity of 81.65 and 65.69%, respectively. In addition, the results also revealed the presence of activation loop motif, ATP binding site, and calmodulin-binding domain of two CDPK proteins (Figures 6A,B), which are considered signature motifs of *CDPK* gene. MEGA7 phylogenetic analysis based on protein sequences showed that CDPK1 and CDPK2 were most closely related to proteins from *Alternaria gaisen* (KAB2111357.1) and *Exserohilum turcicum* (B2M1T6), respectively (Figures 6C,D). Interestingly, the representative motif of CDPK protein varies significantly among different species. For example, the identified calmodulin-binding domain (VKKNFNARRTLHAAIDTIRAINQLRA) of CDPK2 in MG1 was consistent with that in *Arthrobotrys dactyloides*, but completely different from those in *Sporothrix schenckii* (KARLRRGIELVKLTNRIEAL) and *Coprinopsis cinerea* (KDAVAVVRSILS, WKSAIASARALNR) (Tsai et al., 2002; Valle-Aviles et al., 2007; Masashi et al., 2018). However, no calmodulin-binding domain was identified of CDPK1 protein in MG1. In plants, the typical active domain is called EF-hand calcium-binding motifs, which generally has four. They are predominant calcium sensor (Wang and Shao, 2013).

**Figure 7:**
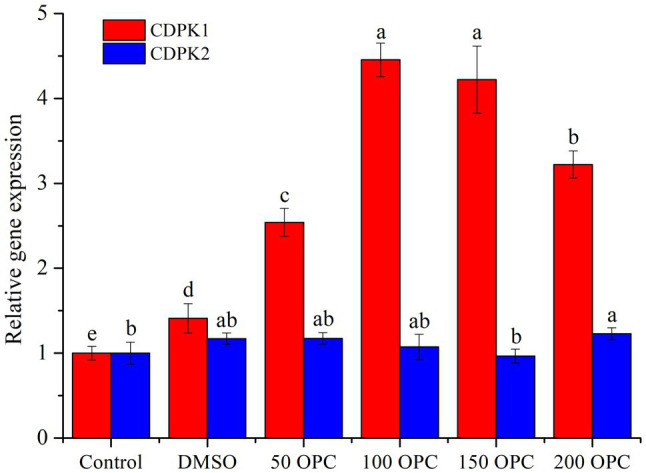
Effect of OPC on the expression level of CDPKs from MG1.

It can be seen from Figure 7 that different concentrations of OPC had no effect on the expression of CDPK2, while the expression of CDPK1 first increased and then decreased with the increase in OPC concentration and reached the maximum at 100μm OPC, which was about 4.5 times that of the control. In addition, the trend of *CDPK1* expression was positively correlated with that of resveratrol production. Therefore, the influence of OPC on the resveratrol biosynthesis in MG1 may be related to the regulation of *CDPK1*, and the specific mechanism needs to be further analyzed. Although little is known about the regulated mechanism of CDPKs in fungi, many of the signal-induced responses have been well documented in controlling spore germination and differentiation, cell division, and protein sorting in the secretory pathway (Tsai et al., 2002).

### Synergistic Effect of UV Light and OPC on the Biosynthesis of Resveratrol in *Alternaria* sp. MG1

**Figure 8:**
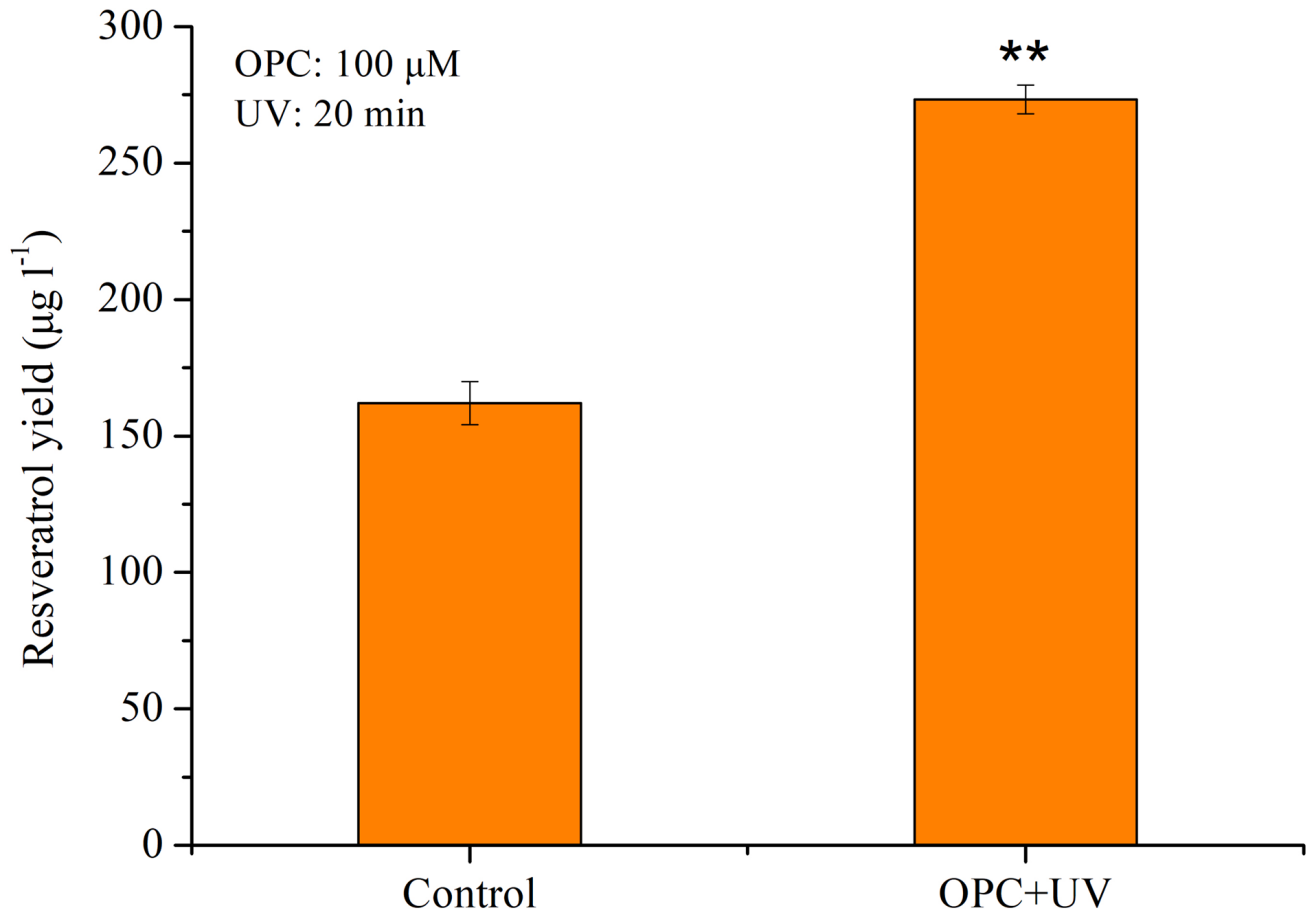
Effect of OPC combined with UV on the resveratrol production, ^**^p<0.001.

To further improve the production of resveratrol, UV light combined with OPC was employed to treat MG1 cells. As shown in Figure 8, synergistic treatment increased the production of resveratrol by 70.37% and finally reached 276.31μg/l. In our previous study, ethanol was found to have a positive effect on resveratrol synthesis due to its role as a precursor and carbon source. It increased the production of resveratrol in a concentration-dependent manner, with the highest increase by 26.31% (Lu et al., 2019). In addition, salicylic acid (SA) and methyl jasmonate (MeJA) were reported to be related to phenylpropane pathway genes toward accumulation of stilbenes and flavonoids. Results showed that SA induction resulted in a 33.32% increase in resveratrol production, whereas MeJA did not affect the resveratrol production nor the expression of related genes. Compared with all single factor treatments, combination treatment of UV light and OPC showed great potential in promoting the biosynthesis of resveratrol in MG1. This strategy is also used to increase the production of resveratrol in other microorganisms. Villa-Ruano et al. (2021) found that citral and Glucanex (mixtures of hydrolytic enzymes) produced up to 225.1mg/l of resveratrol in *Arcopilus aureus* MaC7A, which was significantly higher than that from either citral (187.8mg/l) or Glucanex (198.3mg/l). And, a final production of 237.6mg/l was obtained by combination of tyrosine (200mg/l), citral (50mg/l), thymol (50mg/l), and Glucanex (100mg/l) in batch cultures. Therefore, synergistic effect of multiple elicitors is an effective way to increase the yield of secondary metabolites derived from plants or microbes and is widely employed in other studies. The combination of yeast extract (YE) and Ag+(300 micromol x L(−1)) or and Co2+ (100 micromol L(−1)) led to the highest tanshinone I and tanshinone IIA content, which was nearly 14-fold and 14.5-fold of the control, respectively (Yan et al., 2006). Serial treatment of methyl jasmonate (MJ), salicylic acid (SA), and YE at 24-h intervals enhanced the accumulation of dihydrosanguinarine (2.5 times) and sanguinarine (5.5 times) in *Eschscholtzia californica* suspension cultures (Cho et al., 2008). This strategy was more effective than single elicitors. In addition, the combined elicitors of MJ, jasmonic acid, and chitosan synergistically promoted greater valtrate production than each individual elicitor addition. It was 1.4-fold higher than the maximum increase obtained by individual elicitor addition (10.58mg/g with 100mg/lMJ).

## Conclusion

Our study demonstrated that ultrasound and sodium butyrate had no effect on increasing the resveratrol production and related gene expression, while UV radiation and co-culture with *Phomopsis* sp. XP-8 significantly improved the biosynthesis of resveratrol with the higher yield coming from UV 20min. Co-culture altered the profiles of secondary metabolites of MG1 with 65 new generated and 75 undetected compounds, resulting in the promoting of stilbene synthesis, inhibiting lignin synthesis, and converting naringenin to various downstream flavonoids. OPC, one of the most important nutrients in grape, also showed significant promotion of biosynthesis of resveratrol in MG1, and the induction mechanism was revealed to be related to the regulation of CDPK1 protein. By combination of UV radiation and OPC, the highest production of resveratrol in MG1 was obtained. The study showed great application potential of endophytic fungi for producing high-valuable chemicals and provided an effective strategy for accumulation of resveratrol by unengineered microorganisms.

## Data Availability Statement

The original contributions presented in the study are included in the article/Supplementary Material, and further inquiries can be directed to the corresponding author.

## Author Contributions

YLu, JS, and YLi conceived and designed the experiments. YLu and XZ performed the experiments. YLu and JS wrote the draft manuscript. YS, YQ, and YLi reviewed and edited the manuscript. All authors contributed to the article and approved the submitted version.

## Funding

This work was supported by National Key Research and Development Project (2019YFD1002500), China Agriculture Research System (CARS-29-jg-03), and Chinese Universities Scientific Fund (2452021023), and National Natural Science Foundation of China (32172183).

## Conflict of Interest

The authors declare that the research was conducted in the absence of any commercial or financial relationships that could be construed as a potential conflict of interest.

## Publisher’s Note

All claims expressed in this article are solely those of the authors and do not necessarily represent those of their affiliated organizations, or those of the publisher, the editors and the reviewers. Any product that may be evaluated in this article, or claim that may be made by its manufacturer, is not guaranteed or endorsed by the publisher.
