# Supplementary material for: Improvement of the Biosynthesis of Resveratrol in Endophytic Fungus (Alternaria sp. MG1) by the Synergistic Effect of UV Light and Oligomeric Proanthocyanidins
Source: Front Microbiol. 2021 Oct 21;12:770734. doi: 10.3389/fmicb.2021.770734 (PMC8567136; doi:10.3389/fmicb.2021.770734)
Supplement: Supplementary file 1 [file Data_Sheet_1.DOCX]

**Supplemental File 1**

*CDPK1*

ATGCGCCAACTAGACCACCCCAACATCGTCAAACTTATCGACTTTTCCGAATCACGGCAGTACTACTACATCGTGCTCGAGCTGTGCCCAGGAGGCGAACTGTTCCATCAAATCGTCCGTCTAACGTACTTCTCCGAGGACCTTTCCCGTCACACCATCATTCAGGTGGCCAAGGCTCTGCAGTACCTGCACGAGGAAGCAGGTGTTGTCCATCGAGACATCAAGCCTGAGAACCTCTTGTTCTATCCCACACCATTCATCCCCACACGCAACCCAAAGCCAAGGGGACCAGACGATGAAGACAAGGCTGACGAGGGAGAGTTCGTCAAGGGCAAGGGCGCTGGTGGTATCGGCCTAATCAAGATTGCCGACTTCGGCCTTAGCAAGGTTATCTGGGACACGCAAACCATGACACCTTGCGGTACTGTCGGCTATACTGCACCTGAGATTGTCAAGGATGAGCGTTACTCTAAGAGTGTCGACATGTGGGCGTTGGGTTGTGTGCTTTACACACTTCTCTGTGGTTTCCCGCCGTTCTACGACGAGTCCATCCAAACCCTCACTGAGAAGGTTGCTCGTGGCCAGTACACCTTCCTCTCACCCTGGTGGGACGATATCTCTAAGTCCGCGCAAGACTTGGTCTCCCATCTGCTGACAGTCGACCCGGAAAAGCGATATGACATCAACCAATTCCTCAACCACCCATGGATACGTGAAGCCGATGAGCCAACCTACTCTGCCTACGACGCACCACCCCTCGCCACACCTGCCGCCAAAAAGGAGCGTCTGCAACCAGACTTCTCGCACTTGGAGTCACCGGGTGCTCGTCGCATGGACTTCCGCTCTCCTGGTGCCGTCAACCTCCGAGAAGTATTCGATGTCAGTTATGCTGTACATCGACAGGAAGAGGAGGGCAAGAGGAAGAAGCAGTTCAAGCAGGGTTACCGTGGCGCCAACGCCATGAACTCGTTGAACGCCCTCGATGAGGGTGAAGACGATGACGAGTATGCTGCTGAATCTGTCCCATACGACCCATCATCGCAACATCCACCAGCGAAGCTCCCTAAGTCTCAAGGTGCTGATGTCACTGGTATGGAGCAGAAGATGCGCACCACCACACTATCCGCCGCAGCTCAGGCCCGCCAGCAACAATCAGCACCACGACAGCAAGAGCGAGGATATGGCCAACACTCTCCAGCTGTTGCGGCTGCAGCGAAGCGCCAAGTCAGGAACAAGGGCGCATTTGAGCTCAACATGGACAGCTCGACATTACTCGGTCGTCGAAACAAGAAGGGTCCTGAGCCAAGCGGCTTAAGAAACACAACTACGGTTGGCGGTACATGA

*CDPK2*

ATGGCGACAAGGACTTCGAACGGCGCTTCTGGAGCGCAGGGTACACAGACGGCAGCGCAAGTGCAGCCTTGCAGATACAAGACTGGCAAAACTTTAGGCGCAGGCAGCTACTCTGTAGTGAAGGAGTGCGTACACATTGATACAGGAAGATACTATGCCGCCAAGGTCATCAACAAGCGGCTCATGGCAGGCCGAGAACACATGGTCAGAAATGAGATTGCCGTGCTTAAGAGAGTATCTATGGGTCACAGAAACATTCTGACACTCGTTGATTACTTCGAAACTATGAACAACCTTTACCTTGTCACCGACCTTGCGCTTGGAGGCGAGCTTTTCGACCGCATCTGCCGAAAGGGCAACTATTACGAATCTGACGCTGGTGATCTTATCCGTGCCACGCTTTCTGCTGTCGCATACCTTCACGACCACGGCATCGTACATCGAGACTTGAAGCCTGAGAACCTCTTGTTCCGAACACCAGAAGACAATGCCGATCTGCTGATTGCCGACTTTGGGCTGTCTCGAATTATGGATGAAGAGCAGTTCCACGTCCTGACAACGACATGCGGAACGCCAGGTTACATGGCACCTGAGATCTTCCGCAAAACTGGACACGGCAAACCGGTTGACATATGGGCTATCGGCGTTATCACATATTTCCTCCTCTGCGGTTATACTCCGTTCGACCGCGACTCTAACCTCGAAGAGATGCAGGCCATCTTAGTCGCAGACTACTCCTTTACACCCTTGGAGTATTGGAGGGGTGTTTCTCTTACAGCACGCGAGTTCATTCGTCGCTGTTTGACCGTCGACCCTGCAGCACGCATGACAGCCCATGAAGCCTTGTCTCATCCTTGGATCACGGAGCTTGGCAAGAATAACGCTGATGGTGAAGAGGATCTATTGCCCACGGTCAAGAAGAACTTCAACGCTCGTCGTACCCTGCATGCAGCTATCGATACGATTCGTGCTATCAATCAGCTGAGAGCGGGTGGTGCCGCTGGTATGATGGACGGCCAGCGTTCTGCTGAACCACGGAGAGGTGCCCCTCACGCCAACATCCCTCAGCCAGCTGATGAGCCAGACGACCCCATGGAGATTGACAGTAGAGGAAACGGACACGGTCAGACCGAAGAGATGATACAGGAGCAAGAGCGGAGGATTAGAGAGACCCAGCAAGGCCTGTGGGGTAAGCGATGA
